# Synthesis of europium-doped VSOP, customized enhancer solution and improved microscopy fluorescence methodology for unambiguous histological detection

**DOI:** 10.1186/s12951-017-0301-6

**Published:** 2017-10-10

**Authors:** Angela Ariza de Schellenberger, Ralf Hauptmann, Jason M. Millward, Eyk Schellenberger, Yuske Kobayashi, Matthias Taupitz, Carmen Infante-Duarte, Jörg Schnorr, Susanne Wagner

**Affiliations:** 1 Department of Radiology, Charité-Universitätsmedizin Berlin, Corporate Member of Freie Universität Berlin, Humboldt-Universität zu Berlin, and Berlin Institute of Health, Charitéplatz 1, 10117 Berlin, Germany; 2 Department of Interventional and Diagnostic Radiology and Nuclear Medicine, University Medical Center Hamburg-Eppendorf, Martinistraße 52, 20246 Hamburg, Germany; 3 Berlin Ultrahigh Field Facility, Max Delbrück Center for Molecular Medicine, Robert-Rössle-Str. 10, 13125 Berlin, Germany; 4 Institute for Medical Immunology, Charité-Universitätsmedizin Corporate Member of Freie Universität Berlin, Humboldt-Universität zu Berlin, and Berlin Institute of Health, Augustenburger Platz 1, 13353 Berlin, Germany

**Keywords:** VSOP, Europium, Fluorescence, MRI

## Abstract

**Background:**

Intrinsic iron in biological tissues frequently precludes unambiguous the identification of iron oxide nanoparticles when iron-based detection methods are used. Here we report the full methodology for synthesizing very small iron oxide nanoparticles (VSOP) doped with europium (Eu) in their iron oxide core (Eu-VSOP) and their unambiguous qualitative and quantitative detection by fluorescence.

**Methods and results:**

The resulting Eu-VSOP contained 0.7 to 2.7% Eu relative to iron, which was sufficient for fluorescent detection while not altering other important particle parameters such as size, surface charge, or relaxivity. A customized enhancer solution with high buffer capacity and nearly neutral pH was developed to provide an antenna system that allowed fluorescent detection of Eu-VSOP in cells and histologic tissue slices as well as in solutions even under acidic conditions as frequently obtained from dissolved organic material. This enhancer solution allowed detection of Eu-VSOP using a standard fluorescence spectrophotometer and a fluorescence microscope equipped with a custom filter set with an excitation wavelength (λ_ex_) of 338 nm and an emission wavelength (λ_em_) of 616 nm.

**Conclusion:**

The fluorescent detection of Eu-doped very small iron oxide nanoparticles (Eu-VSOP) provides a straightforward tool to unambiguously characterize VSOP biodistribution and toxicology at tissue, and cellular levels, providing a sensitive analytical tool to detect Eu-doped IONP in dissolved organ tissue and biological fluids with fluorescence instruments.

**Electronic supplementary material:**

The online version of this article (doi:10.1186/s12951-017-0301-6) contains supplementary material, which is available to authorized users.

## Background

The development of iron oxide nanoparticles (IONP) [1–9] as contrast agents for magnetic resonance imaging (MRI) [10, 11] started about 4 decades ago. However, IONP that had early been approved by the US Food and Drug Administration (FDA) for liver imaging such as Endorem/Feridex^®^ (AMAG Pharmaceuticals, Inc., Walthman, MA, USA) [12] and Resovist^®^; (Schering AG-now Bayer Pharma AG, Berlin, Germany) [13] have not been further developed or have been withdrawn from the market [14–16]. IONP was slowed down by the fast establishment of gadolinium-based contrast agents for clinical MRI and first-pass arterial MRI angiography [17]. Recently, the safety concerns raised against some gadolinium-based contrast agents (GBCA) [18] have motivated new attempts at off-label clinical used of magnetic IONP such as ferumoxytol [19, 20]. Initially developed to treat iron anemia [21, 22], ferumoxytol/feraheme^®^ is highly suitable for T1- and T2-weighted MRI. Besides the use of IONP in MRI, the emerging technique of magnetic particle imaging (MPI) requires the development of new superparamagnetic IONP [23]. Therefore, the development of iron oxide-based contrast agents remains a topic of high experimental and clinical relevance.

Very small iron oxide particles (VSOP), with a hydrodynamic diameter of 7 nm, combine high cellular uptake with low cytotoxicity due to their biocompatible citrate coating [24–28]. Earlier versions of these IONP, produced as VSOP-C184 (Ferropharm, Teltow, Germany) [29], were developed to the level of phase I and II as blood pool contrasts agent for MR angiography [30–32], but they are no longer available from the company. In the last decade, the synthesis of VSOP has been refined in our laboratory, and VSOP have been explored for vascular imaging [32, 33] and other applications such as assessment of myocardial inflammation [34], sensitive detection of blood–brain barrier alterations [35], in vivo tracking of monocyte migration after intracerebral injection [36], and discrimination of different inflammatory events in an animal model of multiple sclerosis [37]. Early studies recognized VSOP accumulation in atherosclerotic lesion, 1 h after IV injection in rabbits [38], as opposed to 3 days required for accumulation of ferumoxytol in atherosclerotic lesions, in hyperlipidemic rabbits [39]. Recently, the potential of VSOP [38, 40–43] and other IONP for atherosclerotic plaques characterization has been explored [44, 45].

Further developed IONP such as VSOP require detection tools for their in vitro characterization, e.g., tissue localization, biodistribution, toxicology, and stability. Bimodal contrast agents for MR and fluorescence imaging are commonly produced by functionalization of the IONP coating with fluorescent dyes [46–48]. However, a major drawback of this method, especially for small IONP like VSOP, is the possibility that modifications of IONP coating could alter the particle’s pharmacokinetic bahavior, cause in vivo IONP degradation, and lead to loss of the fluorescent dye [49].

An alternative to functionalization of the IONP coat is iron oxide core functionalization by intercalation of lanthanide ions for example [50] and detection of the intercalated lanthanide to identify the localization of IONP in tissues. A method of magnetite core dotation with lanthanides was first described for dextran-coated ultrasmall mixed ferrite iron oxides (USMIOs) [51].

Here we used europium (Eu) to intercalate it into the core of VSOP to develop Eu-VSOP. Europium is a lanthanide (Ln) that does not occur physiologically and is not used in drugs, and therefore is not, normally present in biological systems. Early europium toxicological studies in rats showed that europium salts had no effect on rat growth, complete blood count, or organ function after being included in the food at concentrations of up to 1% EuCl_3_ over 12 weeks [52, 53]. However, possible Eu release from VSOP needs to be ruled out to avoid safety issues as currently discussed for gadolinium [54–57]. VSOP without europium doping should be used in future clinical applications. Furthermore, the direct excitation of lanthanides for fluorescent detection is ineffective, because the 4f–4f electronic transitions in Ln^3+^ ions are forbidden by the electronic dipole selection rules, leading to very low molar extinction coefficients. A solution to this is the use of an antenna system—an organic chromophore and chelator—that coordinates the Ln^3+^ ion, changing the symmetry of the orbitals, and transfers the energy required for fluorescence excitation to the lanthanide [58]. Additionally, europium chelates exhibit large Stokes shifts (280 nm) and narrow emission spectra, which ensure optimal fluorescent detection with high specificity.

We recently reported accumulation of Eu-VSOP in atherosclerotic plaques of an ApoE^−/−^ mice model, highlighting the relevance of unambiguous detection of iron oxide nanoparticles. Detection of these IONP in tissues is routinely done by iron staining (Perl’s stain), which, however, does not consistently discriminate intrinsic iron from IONP iron (VSOP) [41]. In that study, we showed unambiguous detection of Eu-VSOP in tissues by quantitative spatial detection of europium using laser ablation inductively coupled plasma mass spectrometry (LA-ICP-MS) [59]. Previously, our group demonstrated higher sensitivity of Eu-VSOP fluorescence detection in solution compared with the phenanthroline iron detection method [60]. We present the complete synthesis of citrate-coated very small iron oxide nanoparticles and the procedure for stably intercalating Eu^3+^ into their iron oxide core to produce Eu-VSOP. In addition, we prepared a customized enhancer solution (histo-Eu-enhancer) with neutral pH and increased buffer capacity, and improved the fluorescence methodology to achieve sensitive and stable detection of Eu-VSOP in cells and histological tissue sections. Overall, we show here that Eu-VSOP can now also be imaged by fluorescence microscopy and quantitatively determined by widely available fluorescence spectrophotometers.

## Methods

### Nanoparticle synthesis

Synthesis of the citrate-coated very small iron oxide nanoparticle (VSOP) and europium-doped citrate-coated VSOP (Eu-VSOP) was performed according to the method of Goodarzi et al. [61].

#### VSOP

27.3 g (100 mmol) iron(III) chloride hexahydrate (Sigma-Aldrich, USA) and 14.0 g (70 mmol) iron(II) chloride tetrahydrate (Sigma-Aldrich, USA) were successively dissolved in water (200 mL) at 0 °C. Ammonia solution (90 mL) (28.0–30.0%, Sigma-Aldrich, USA) was added under vigorous stirring. The resulting black suspension was stirred for further 30 min at 0 °C. After magnetic separation, the supernatant was removed, leaving a residue of 100 mL. 300 mL of citric acid (Sigma-Aldrich, USA) solution c(C_6_H_8_O_7_) = 0.348 mol/L was added under stirring and heated to 80–90 °C for 60 min. After a further magnetic separation, the supernatant was separated, centrifuged, washed, and concentrated to 100 mL by ultrafiltration with a 100 kD (PES) Vivaflow filter (Sartorius, Göttingen, Germany) until the filtrate was colorless. Then 100 mL water and 1 mL of a saturated sodium chloride (Merck, Germany) solution was added and the pH adjusted to 5.5 with citric acid. The solution was again concentrated to 100 mL and washed with water until the conductibility of the filtrate was lower than 10 μS. This procedure was repeated three times, and finally the solution was concentrated to 50 mL.

#### Dotation of VSOP with Europium (Eu-VSOP-1 to Eu-VSOP-7)

Different amounts of europium(III) chloride hexahydrate (Sigma-Aldrich, USA) were added to synthesized VSOP (0.236 g for Eu-VSOP-1; 0475 g for Eu-VSOP-2; 0.706 g for Eu-VSOP-3; 0.880 g for Eu-VSOP-4; 1.185 g for Eu-VSOP-5; 1.77 g for Eu-VSOP-6; and 2.37 g for Eu-VSOP-7). The europium chloride needs to be dissolved after the iron(III) chloride has been dissolved to ensure sample homogeneity. Once the europium(III) chloride is completely dissolved, the iron(II) chloride can be added.

### Chemical characterization of the nanoparticles

#### Nanoparticle iron quantification

The iron concentration of synthesized Eu-VSOP was determined using the phenanthroline method [62, 63]. In short, 5 mL hydrochloric acid (Sigma-Aldrich, USA) (c(HCl) = 6 mol/L) were added to 0.5 mL of the nanoparticle solution and dissolved under heating (60 °C) for 20 min. The solution was then diluted to a total of 50 mL with water (stock solution). 1 mL of the stock solution was diluted to a total of 25 mL with water. The diluted solution (2 mL) was used to develop the phenanthroline reaction by mixing it with 1 mL of hydroxylamine hydrochloride (Sigma-Aldrich, USA) solution w(NH_2_OH·HCl) = 10% and 7 mL of the phenanthroline hydrochloride solution (1.0 g 1,10-phenanthroline hydrochloride (Merck, Germany), 14.0 g acetic acid w(CH_3_COOH) = 99–100% (Sigma-Aldrich, USA) and 21.7 g sodium acetate trihydrate (Carl Roth, Germany) dissolved in water to 1 L). The developed colorimetric reaction was photometrically measured after 15 min incubation at room temperature (RT) at 510 nm in a SPECORD 205 spectrophotometer (Analytik Jena, Germany) using the Win ASPECT software (Version 2.1.1.0).

As reference, 2 mL of iron standard solution (1000 mg/L ± 0.2%, Carl Roth, Germany) were diluted with water to 100 mL. This diluted solution (2 mL) was mixed with 1 mL of hydroxylamine hydrochloride solution (w(NH_2_OH·HCL) = 10%) and 7 mL of the phenanthroline hydrochloride solution and also measured at 510 nm.

#### Nanoparticles size

Nanoparticles size was measured by dynamic light scattering at a Zetasizer Nano ZS (Malvern Instruments Ltd., Worcestershire, UK) equipped with the Zetasizer Software Version 6.20. The samples were diluted with HEPES (Sigma-Aldrich, USA) solution c(HEPES) = 10 mmol/L (pH = 7.4) to a final iron concentration of 1 mmol/L.

**Fig. 1:**
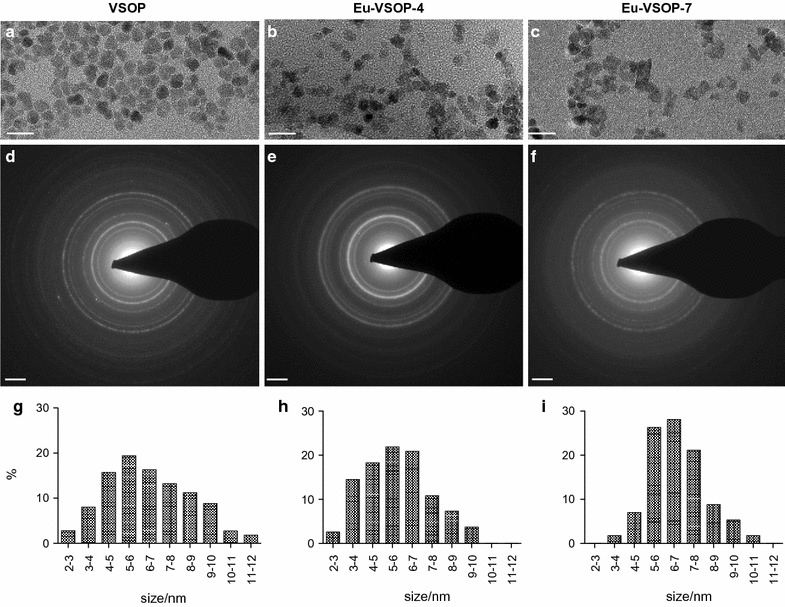
Transmission electron microscopy (TEM) images show monocore structure and mean iron core sizes of approx. 5–7 nm for VSOP (**a**), Eu-VSOP-4 (**b**), and Eu-VSOP-7(**c**). Selected areas of electron diffraction (SAED) pattern (**d**–**f**) confirms the magnetite/maghemite structure and percentage of IONP as distributed by size (**g**–**i**). Scale bar **a**–**c**: 20 nm; scale bar **d**–**f**: 2 nm^−1^

Mean iron core sizes and magnetite/maghemite structure of IONP was confirmed by transmission electron microscopy (TEM). TEM examinations were performed by the Zentraleinrichtung Elektronenmikroskopie (ZELMI) at the Technical University Berlin. High-resolution transmission electron microscopy (HRTEM) using a TECNAI G2 20 S-Twin (FEI-Company, Hillsboro OR, USA) was used with accelerating voltage of 200 kV and a 300 mesh Cu-grid (Fig. 1).

#### Magnetic characterization of VSOP and Eu-VSOP

All measurements were performed at 40 °C and 40 MHz (0.94 T) using an MR spectrometer (Minispec mq 40; Bruker Karlsruhe, Germany). Diluted nanoparticle solutions with iron concentrations between 0.1 and 1.5 mmol/L were prepared. Three solutions with different concentrations were measured for each sample. The relaxation coefficients R_1_ and R_2_ were obtained by linear fitting of T_1_ and T_2_ relaxation rates, and values were normalized to the iron concentrations.

### Enhancer solution for histological use

Enhancer solutions following the principle described by Hemmilä et al. [64] are commercially available for fluorescent detection of Europium with analytical purposes.

We tested the DELFIA^®^ enhancer solution (Perkin Elmer) (pH 2.5) but found it to be of limited use for detecting the fluorescent signal of Eu-VSOP, and therefore we produced a customized enhancer solution with improved buffer capacity (pH 6.6) to allow the detection of Eu fluorescence under physiological pH conditions and a comparative study is presented below.

The customized histo-Eu-enhancer (HEE) was prepared with the same antenna system as used by the DELFIA^®^ enhancer solution (Perkin Elmer) with a modified dihydrogen phosphate-hydrogen phosphate buffer system. This antenna is formed by the ligands β-NTA and TOPO molecules that coordinate the europium ion localized in the core. This complex is surrounded by Triton X-100, forming a micelle-like structure (Additional file 1: Figure S1).

To prepare the solution, first 8.640 g (0.072 mol) sodium phosphate monobasic (Sigma-Aldrich, USA) and 7.506 g (0.053 mol) sodium phosphate dibasic (Sigma-Aldrich, USA) were dissolved in 600 mL water, which was followed by addition under stirring of 1.25 mL Triton X-100 (Sigma-Aldrich, USA), 5 mg (18.8 μmol) 4,4,4-Trifluor-1-(2-naphthyl)-1,3-butadione (β-NTA) (Sigma-Aldrich, USA), and 24.2 mg (62.5 μmol) Tri-n-octylphosphine oxide (TOPO) (Sigma-Aldrich, USA). The mixture was sonicated at 600 W for 30 min, filled up to 1 L and sonicated once more for 100 min. This resulted in a modified enhancer solution termed ‘histo-Eu-enhancer’ (HEE) with a pH of 6.6.

### Quantification of europium fluorescence

All fluorescence acquisitions were performed on a Hitachi fluorescence spectrometer F-7000 with 338 nm excitation wavelength and 616 nm emission wavelength using a photo multiplier tube (PMT) at 400 V and a slit of 10 nm.

**Fig. 2:**
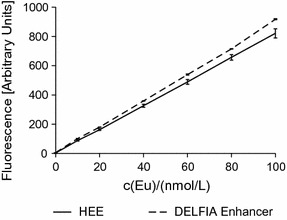
Quantification of Eu fluorescence. Eu fluorescence signal intensity increased linearly with increasing Eu concentration with both enhancer solutions. Therefore, both systems are suitable for quantitative detection of Eu^3+^ in solutions with neutral pH (n = 5). Regression analysis for HEE (r^2^ = 0.9999) and for DELFIA^®^ (r^2^ = 0.9994)

Eu fluorescence intensity was quantified with both enhancers using a Europium(III) standard solution (1001 ± 5 mg/L, Fluka, Swiss) diluted in water at a concentration range between 100 and 1000 nmol/L and mixed with the HEE (1:9) (Fig. 2). This solution was also used for calibration curves required to validate measurements and compensate for variations which, as we observed, are unavoidable between devices and are also due to changes in the excitation lamp intensity. This calibration curve was used to determine the europium content of the synthesized Eu-VSOP. Therefore, the Eu-VSOP were dissolved in hydrochloric acid c(HCL) = 6 mol/L and then diluted with water until the europium concentration was between 100 and 1000 nmol/L. The diluted solution (0.1 mL) was mixed with 0.9 mL of the enhancer solution and fluorescence was assessed as described.

**Fig. 3:**
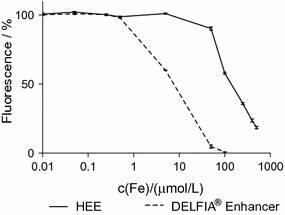
Stability of Eu fluorescence in presence of free iron(III) ions. The fluorescence intensity developed by each enhancer solution was set to 100% for a solution with constant c_final_(Eu) = 50 nmol/L and without iron(III). The fluorescence signal intensity developed by DELFIA was reduced to 10% when exposed to c(Fe) > 1 µmol/L while the Eu fluorescence intensity developed with HEE remained at 100% in the presence of c(Fe) up to 100 µmol/L. The SD of the mean fluorescence signal (n = 3) for HEE variates between 0.7 and 8% and between 0.3 and 11% for DELFIA^Ⓡ^

The stability of the fluorescent compound (enhancer-Eu-VSOP) is pH-dependent as described by Hemmilä [64]. Therefore, we performed Eu fluorescence quantification in the presence of free ions present in organic solutions such as Ca^2+^ (not shown) and Fe^3+^. Several Eu^3+^ solutions with a constant concentration of Europium c(Eu) = 500 nmol/L and different Fe^3+^ concentrations in the range of 0.1 to 10,000 μmol/L were prepared. 100 μl of these solutions were mixed with 900 μL of the enhancer solution, and fluorescence intensity measured with both enhancer solutions was compared for three independent samples (Fig. 3).

**Fig. 4:**
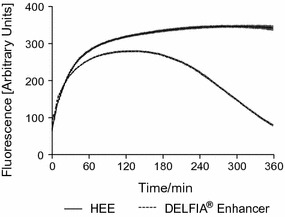
Fluorescence of Eu-VSOP. Fluorescence intensity of doped Eu-VSOP-7 (60 nmol/L) increased over time (measured every 2 min for 6 h; n = 3) with the HEE solution, while it drastically decreased after ~ 2 h with the DELFIA^®^ enhancer solution. The SD of the mean fluorescence signal for HEE variates between 0.8 and 9.4% and between 0.84 and 5.86% for DELFIA^®^

The fluorescence intensity of intact Eu-VSOP-7 was quantified independently with both enhancer solutions (HEE or DELFIA^®^) and acquired every 2 min for 6 h for three different samples (Fig. 4).

### Cellular uptake of Eu-VSOP

The macrophage cell line RAW 264.7 (ATCC Cell Biology Collection (Promochem LGC, Molsheim, France), derived from mice peritoneal macrophages and transformed by the AMLV (Abelson Murine Leukemia Virus), was used. Cells were cultured in Dulbecco’s Modified Eagle’s Medium (DMEM, ATCC), supplemented with 10% of fetal bovine serum (FBS, Gibco) and 1% penicillin–streptomycin (penicillin 10,000 units/mL, streptomycin 10 mg/mL; Sigma-Aldrich), at 37 °C in a 5% carbon dioxide humidified atmosphere. Cells were regularly passaged before reaching confluence with medium change every 2 days.

To achieve cellular uptake of Eu-VSOP, macrophages were seeded at 40,000 cells/cm^2^ in six-well plates with growth medium (1.9 mL/well). The next day, cells were incubated for 24 h. Therefore, growth medium was replaced by IONP solution at 0.5 mmol/L iron concentration (VSOP, Eu-VSOP-3, or Eu-VSOP-7) in DMEM without phenol red and 1% fetal bovine serum (FBS, Gibco) for cell synchronization [65]. Negative controls (empty cells) were prepared under the same conditions but in the absence of nanoparticles.

**Fig. 5:**
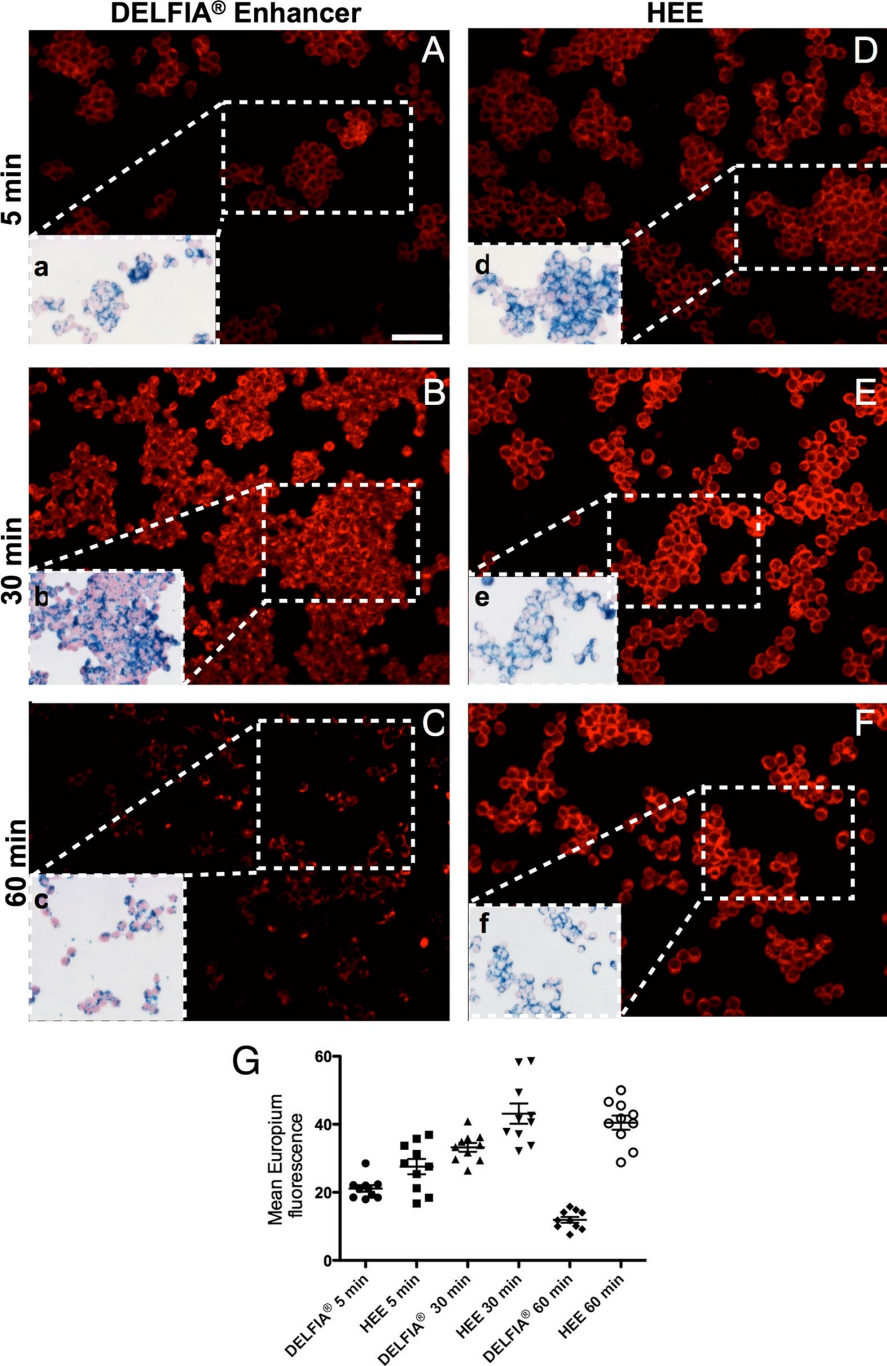
Fluorescent signal of Eu-VSOP in cells. Eu-fluorescent signal was successfully detected at 615/20 nm in macrophages labeled with Eu-VSOP-7. Eu-fluorescence developed with customized HEE increased after 30 min, and remained similar after 60 min (**E**, **F**); while Eu-fluorescence obtained with DELFIA^®^ enhancer was lower at 5 min, similar at 30 min, but significantly reduced after 60 min (**A**–**C**). This is corroborated by semiquantitative analysis of Eu fluorescence intensity for 10 regions of interest (ROI) done with ImageJ (**G**). P < 0.001 two-way ANOVA. Scale bar: 100 µm

All cells were washed 3 times with PBS and passaged to remove nanoparticles that attached to plastic surfaces of the culture plates. For fluorescence assessment of europium, cells were passaged into adherent chamber slides (Thermo Fisher Scientific, Waltham, MA, USA) at 4000 cells/cm^2^ for fluorescence microscopy (Fig. 5).

For population doubling time (PDT) quantification, cells were passaged into six-well plates at 1000 cells/cm^2^ with complete growth medium and incubated for 8 days with medium exchange every 2 days. Every 2 days, cells were counted to determine the population doubling time (PDT = T*n2/ln (A/A_0_): T = time between cell counts, A = final cell number, A_0_ = initial cell number) (Additional file 2: Figure S3).

### Animal experiments

All experimental procedures were approved by the regional animal study committee of Berlin, the Landesamt für Gesundheit und Soziales Berlin (LAGeSo), and mice were acquired and handled in accordance with the guidelines published in the NIH Guide for the Care and Use of Laboratory Animals (NIH Publication No. 85–23, revised 1985). C57BL/6 mice were bred and maintained in the facilities of the “Forschungsinstitut für Experimentelle Medizin” (FEM, Charité—Universitätsmedizin, Berlin, Germany) under specific pathogen-free conditions. Eu-VSOP was administered intravenously to 6–8-week-old female mice at a dose of 0.2 mmol/kg. After 24 h, mice were processed for histology. Following terminal anesthesia, mice were transcardially perfused with 20 ml PBS, then with 20 mL zinc fixation solution (0.5% zinc acetate, 0.5% zinc chloride, 0.05% calcium acetate). Spleens were extracted and subsequently fixed in diluted zinc solution (1:10) for 3 days at room temperature.

### Fluorescence detection of Eu-VSOP in cells and tissue sections

Cells grown in chamber slides were washed 3 times with PBS before fixation with − 20° pre-cooled acetone-methanol (1:1) for 20 min. Fixation solution was removed and wells were air-dried for 5 min. Pre-cooled enhancer solutions were kept at 4° in the dark. Either the DELFIA^®^ enhancer solution (PerkinElmer, Germany) or our customized HEE was added to the cells and incubated in the dark for 10 min at RT. The enhancer solutions were removed, slides were air-dried for 5 min in the dark and mounted with cover slips and Fluoromount^TM^ mounting medium (Sigma-Aldrich Co, St Louis, MO, USA). Photomicrographs were taken 5 min, 30 min, and 60 min later. Identical settings, e.g., light exposure time (10 s), were used for all chamber slides. Positions of fluorescence photomicrographs were saved using the Zeiss microscope software for each slide and relocalized to determine iron co-localization of Eu-VSOP after Prussian blue stain (Fig. 5).

**Fig. 6:**
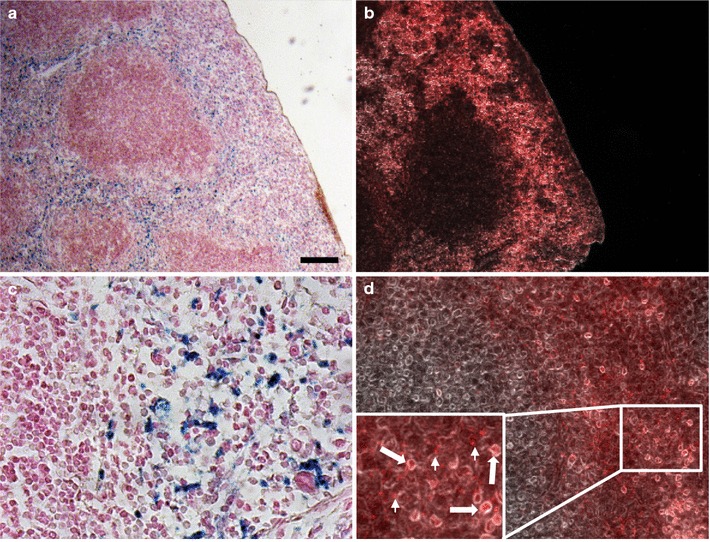
Fluorescence detection of Eu-VSOP in tissue sections. Ex vivo detection of Eu-VSOP in mouse spleen. 24 h after IV administration of Eu-VSOP, iron (blue) was detected in splenic red pulp (**a**, **c**, Prussian blue stain with nuclear fast red counterstain). Serial section with fluorescence detection of europium with HEE (**b**,** d**, red staining, bright-field background). Inset in **d** shows intracellular and extracellular Eu-VSOP accumulations (arrows and arrowheads, respectively). Scale bar: 100 µm

Spleen tissue was dehydrated and embedded in paraffin blocks according to standard procedures, and 5-μm sections were cut on a microtome. Tissue sections were then deparaffinized and re-hydrated. Fluorescent detection of Eu-VSOP was performed after tissue fixation and enhancer incubation as done with cells. Photomicrographs were taken (see below) after enhancer removal and 60 min storage at RT in the dark as done with cells (Fig. 6).

### Iron quantification in cells and tissue sections

Conventional detection of Eu-VSOP by Prussian blue staining (Perl’s method) was done by incubation of slides with 1% potassium hexacyanoferrate (II) solution for 5 min and 1% potassium hexacyanoferrate with 1% HCL (20 min), followed by counterstain with nuclear fast red solution as described [66].

### Microscopy and image analysis

Photomicrographs were obtained in an Axio Observer.Z1 with AxioVision Software ZEN 2012 (Carl Zeiss AG. Oberkochen, Germany). Europium was detected with a customized filter set consisting of an excitation filter (BP 350/50 nm), a beam splitter filter (380 nm LP), and an emission filter (HC 615/20 nm) (AHF Analysentechnik AG, Tübingen, Germany).

Several photomicrographs were taken for repeated cell uptake experiments (n = 5), exemplary photomicrographs are shown (Fig. 5).

Chamber slides with RAW 264.7 cells used for fluorescent detection of Eu-VSOP were washed overnight in Millipore water (mH_2_O) to carefully remove cover slides and subsequently stained for iron. Photomicrographs were taken using the coordinates stored by the microscope software. A slight difference in size between fluorescence and light microscopy images is due to the different resolution of fluorescence and light cameras (Fig. 5). Tissue sections were stained following the same protocol, and consecutive spleen slides were used for fluorescence and conventional iron detection of Eu-VSOP (Fig. 6).

ImageJ (National Institutes of Health, USA) was used to compare fluorescence intensity between photomicrographs (ROIs: n = 10 per time point). White background was set to equal for all images to define regions of interest (ROI) for assessing mean fluorescence intensity (Fig. 5G).

Europium fluorescent signal intensity achieved with both enhancer solutions over time (5, 30, 60 min) was compared by two-way ANOVA analysis using the Graphpad Software (GraphPad Prism 5).

## Results

### Synthesis and properties of the VSOP and Eu-VSOP

Nanoparticle solutions of VSOP and Eu-VSOP prepared using the method described above are suspensions that remain stable for more than 2 years without precipitation when autoclaved and stored in sealed dark glass bottles (data not shown).

The amount of europium intercalating into the iron cores of VSOP (m(Eu):m(Fe) ratio) increased with the amount of Eu used in the synthesis. Thus, Eu-VSOP-7 showed the highest amount of intercalated europium with m(Eu):m(Fe) = 0.0274, which was 3.75 times higher than that achieved for Eu-VSOP-1 with m(Eu):m(Fe) = 0.0073 (Table 1).

**Table 1:** Efficiency of europium intercalation in VSOP

	m(Eu):m(Fe) used in the synthesis [%]	m(Eu):m(Fe) found in the particle [%]
Eu-VSOP-1	1.02	0.73
Eu- VSOP-2	2.05	1.25
Eu- VSOP-3	3.05	1.62
Eu- VSOP-4	3.79	1.88
Eu- VSOP-5	5.12	2.10
Eu- VSOP-6	7.65	2.18
Eu- VSOP-7	10.21	2.74

Transmission electron microscopy (TEM) images of VSOP, Eu-VSOP-4, and Eu-VSOP-7 confirmed the magnetite/maghemite structure and IONP mean iron core sizes of approx. 5 to 7 nm (Fig. 1).

Hydrodynamic diameters assessed by dynamic light scattering revealed a narrow distribution with mean values between 9.9 ± 2.1 nm for all Eu-VSOP and 10.8 ± 2.8 nm for VSOP without intercalated Eu (Additional file 3: Table S1).

Furthermore, the magnetic properties (R1, R2 and MS) of VSOP decreased with increasing amounts of intercalated Eu^3+^ (Table 2).

**Table 2:** Magnetic properties of the synthesized nanoparticles

	R_1_ (L/mmol s)	R_2_ (L/mmol s)	M_S_ (Am^2^/kg Fe)^−1^
VSOP	32	85	103.6
Eu-VSOP-1	28	74	96.5
Eu-VSOP-2	24	61	82.7
Eu-VSOP-3	24	62	86.9
Eu-VSOP-4	28	75	83.5
Eu-VSOP-5	24	65	82.2
Eu-VSOP-6	17	46	59.9
Eu-VSOP-7	19	53	69.6

Magnetic saturation (Ms) indicates a high degree of crystallinity but, along with magnetic relaxivity (R_1_ = T_1_-relaxivity, R_2_ = T_2_-relaxivity), decreases with rising Eu^3+^ content

### Quantification of europium

We observed that the fluorescence intensity developed by both the DELFIA^®^ and customized HEE enhancer solutions with free Eu^3+^ ions is linear to the Eu concentration [c(Eu)] and allows similar detection with free Eu^3+^(Fig. 2).

Comparison of the fluorescence intensity developed by both enhancer solutions for a fixed amount of Eu^3+^ and increasing amounts of free Fe^3+^ ions revealed that the fluorescent signal rapidly decreased to 10% in the presence of c(Fe) higher than 1 µmol/L when the DELFIA^®^ enhancer (pH 2.5) was used. In contrast, the use of HEE resulted in stable fluorescence intensity (100%) with c_Fe_ up to 100 µmol/L (Fig. 3). The standard deviation of the mean fluorescence signal is the highest (~ 8% for HEE and 11% for DELFIA^®^ Enhancer) at the higher iron concentrations due to reducing fluorescence intensity values.

The fluorescence intensity developed with both enhancers for Eu-VSOP-7 was time-dependent. The Initial fluorescence intensity achieved with both enhancer solutions for Eu-VSOP-7 (Fig. 4) was lower than that achieved with free Eu ions at the same c(Eu) (60 nmol/L) (Fig. 2).

The maximum fluorescence intensity achieved with Eu-VSOP-7 using DELFIA^®^ enhancer solution was observed after approximately 110 min and decreased continuously after approximately 2 h over the following 6 h. In contrast, the use of HEE resulted in a constant increase in fluorescence intensity for the entire 6-h period (Fig. 4).

The standard deviation of the mean fluorescence signal is higher (~ 9.4% for HEE and 5.9% for DELFIA^®^ Enhancer) at the beginning of the reaction (up to 10 min) and is reduced at increasing reaction time (up to ~ 0.8% for both enhancers). This might be due to the kinetic of the reaction between the intercalating Eu^3+^ and the antenna molecule of the enhancer.

### Fluorescence detection of Eu-VSOP in cells and tissue

Phagocytic RAW 264.7 macrophages efficiently take up both, VSOP and europium-doped Eu-VSOP. Uptake was observed to increase with VSOP loading concentrations (not shown) and incubation times (Additional file 2: Figure S3B, D). Ten-fold higher IONP uptake (10–13 pg Fe/cell) was reached for VSOP, Eu-VSOP-4, and Eu-VSOP-7 after 24 h incubation in comparison to 4 h incubation of an identical IONP incubation concentration (c_Fe_ = 0.5 mM). Four days after IONP uptake, labeled cells showed significantly increased PDT in comparison with non-labeled cells (Additional file 2: Figure S3C). However, similar PDT was observed 2 days after cell labeling despite a higher concentration of c_Fe_/cell than at day 4. Furthermore, the effect on PDT disappeared after complete dilution of the IONP load (Fe/cell) due to cell division (days 6 and 8) (Additional file 2: Figure S3D).

RAW 264.7 macrophages labeled during 24 h with Eu-VSOP-7 were used to assess the fluorescence intensity signal of Eu-VSOP.

Fluorescence detection of Eu-VSOP was successful with cells, 10 min after incubation of Eu-VSOP-labeled cells with DELFIA^®^ and with the customized enhancer solution (HEE). However, fluorescence intensity increased with HEE over time (5, 30, and 60 min) after removal of the enhancer solution and mounting of the slides with cover slides at RT (Fig. 5). DELFIA^®^ and HEE enhancers reached similar fluorescent signals after 30 min, while the fluorescence intensity of cell slides treated with DELFIA^®^ decreased after 60 min (Fig. 5C, G). In contrast, the fluorescent signal developed with the customized HEE was stable when slides were stored in the dark at RT for more than 60 min and at − 20 °C for at least 2 weeks (data not shown). Conventional detection of Eu-VSOP by iron stain (Fig. 5A–F) confirmed colocalization of iron and fluorescent signals of Eu-VSOP-labeled cells and tissues, but the fluorescent signal of Eu-VSOP appears to be more sensitive than that of the iron stain.

Fluorescent signal measurements of 10 ROIs per image over time, with both enhancer solutions, confirmed that the Eu-fluorescent signal peaks after 30 min and remains stable until 60 min when the slides were incubated with our customized HEE and kept in the dark at RT (Fig. 5G).

To test the detectability of the Eu-fluorescent signal in histological spleen tissue sections, mice were intravenously injected with 0.2 mmol Fe/kg of Eu-VSOP-7 and tissues were dissected 24 h later. This is a typical dose and protocol used in our previous studies with VSOP [35, 37, 67]. When administered i.v., the particles are readily taken up in the spleen.

In spleen tissue sections, the fluorescence detection of Eu-VSOP was possible for both paraffin-embedded (Fig. 6) and cryosectioned tissue (not shown). Within the spleen, the particles accumulated in the reticuloendothelial structures of the splenic red pulp and are largely excluded from the lymphoid follicles, as seen by conventional Prussian blue staining for iron detection (Fig. 6a, c). Adjacent tissue sections processed for fluorescence detection of Eu-VSOP show a corresponding robust europium signal in the red pulp, with relative absence of signal in the lymphoid follicles (Fig. 6b, d). Higher magnification (inset in Fig. 6d) shows Eu-VSOP accumulations that appear to be intracellular (arrows) as well as extracellular (arrowheads) compared to the bright-field image.

## Discussion

### Properties of the VSOP and Eu-VSOP

Coprecipitation of iron(II) and iron(III) chloride with ammonia for VSOP or additionally europium(III) chloride for Eu-VSOP is a straightforward and economic method to synthesize stable citrate-coated iron oxide nanoparticles without or with Eu doping.

As expected, with increasing amounts of Eu used in the synthesis, the total amount of intercalated Eu increased, while both the efficiency of Eu intercalation and the yield of magnetic particles decreased (Table 1).

Consistent with published reports on other particles [68], Eu-VSOP with high europium doping showed reduced magnetic properties (Table 2). Likely, increasing Eu concentrations during synthesis (Eu-VSOP-7) increases the proportion of VSOP with very high Eu content with reduced magnetism which are consequently lost during the magnetic separation steps.

In contrast, the hydrodynamic diameter of Eu-VSOP was not influenced by Eu-doping. The iron oxide core size of approx. 5 to 7 nm is in the same range as for VSOP without Eu-doping, but slightly smaller than the hydrodynamic diameters (in average 11 nm) as the thickness of the citrate coating, including the associated water molecules, is around 1.5 nm [31]. The selected areas of electron diffraction (SAED) pattern confirmed that Eu-VSOP maintained the cubic structure of maghemite/magnetite characteristic of magnetic IONP [68]. Overall, europium doping did not lead to fundamental structural changes of VSOP. This observation is consistent with previous reports on the doping of different IONP with various lanthanides in solvothermal synthesis [69].

The magnetic saturation (Ms) of VSOP indicates a high degree of crystallinity [11] and decreases with rising europium content (Table 2). This might be attributable to distortion of the crystal lattice by the europium ions, which have a significantly larger radius than Fe^3+^ ions (r(Eu^3+^) = 0.95 Å, r(Fe^3+^) = 0.55 Å). The reduced magnetic saturation led to decreased magnetic relaxivity (R_1_ and R_2_) after intercalation of lanthanides into the core of IONP as previously observed by others [51, 70]. Eu-VSOP-4 had the highest magnetic relaxivity of the synthesized Eu-VSOP in the current study. Although our previous in vivo experiments demonstrated Eu-VSOP-7 to have sufficient magnetic properties to produce enough signal for single-cell detection using T2*-weighted MR imaging [66], we suggest to test Eu-VSOP-4 in future applications.

Furthermore, VSOP with comparable magnetic properties have been detected by T2*-weighted MRI in atherosclerotic plaques after nanoparticle accumulation [41, 43].

### Fluorescence of Eu-VSOP

The europium fluorescent signal is detected by using an antenna system provided by the enhancer solution. As described by Hemmilä et al., in the case of free Eu^3+^ ions, each Eu^3+^ is coordinated by three β-NTA and two TOPO molecules surrounded by Triton-X100 forming a micelle structure (Enhancer-Eu^3+^) [64] (Additional file 1: Figure S1).

**Fig. 7:**
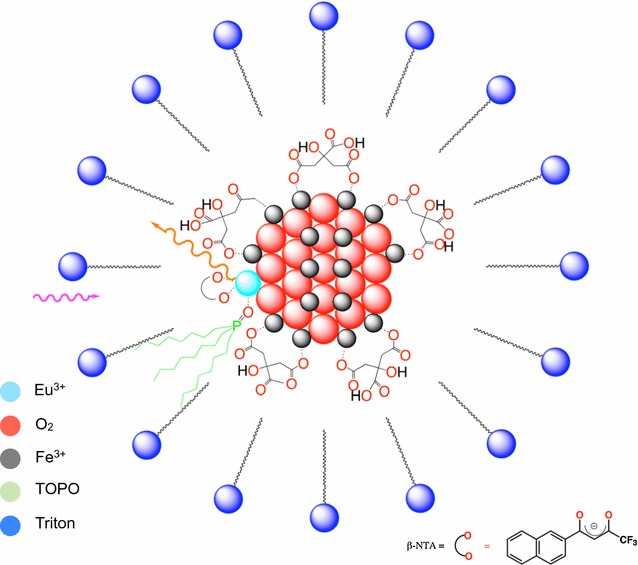
Structure of the fluorescent compound (enhancer-Eu-VSOP). The Eu^3+^ ions located on the surface of the iron core of Eu-VSOP are coordinated by the enhancer components. The HEE is prepared based on the antenna system formed by the chelator (β-NTA) and the ligand (TOPO) with a modified buffer system. This complex is surrounded by Triton X-100 forming a micelle. The suggested structure is based on the structure of commercial enhancers for fluorescence detection of Eu in analytical procedures as suggested by Hemmilä et al.

For energy transfer from the antenna to Eu^3+^ ions on the surface of the iron oxide core of Eu-VSOP, the Eu^3+^ ions must also be coordinated by β-NTA. Therefore, we suggest that a similar micellar structure might be formed to protect the europium ions from quenching by water molecules and propose a similar micelle structure for the fluorescent compound formed with the customized solution (Enhancer-Eu-VSOP) (Fig. 7).

Experiments comparing DELFIA^®^ and the customized HEE enhancer showed a linear relation between the concentration of Eu^3+^ ions and fluorescence intensity (Fig. 2). Therefore, both enhancer systems are suitable for analytical quantification of Eu^3+^ ions. However, the customized HEE has the advantage of high buffer capacity (Additional file 4: Figure S2), which is important for the investigation of organic material that frequently needs to be dissolved in strong acidic solutions.

The Eu fluorescence intensity developed with the commercial DELFIA^®^ enhancer was reduced by free Fe^3+^ ions exceeding 1 μmol/L. In contrast Eu fluorescence intensity with HEE remained similarly stable in the presence of up to 100 μmol/L Fe^3+^ (Fig. 3). Decreased Eu fluorescence intensity using DELFIA^®^ might be attributable to competition between europium and iron ions for the chelator (β-NTA). The equilibrium between these reactions is pH-dependent and higher pH seems to favor the formation of the europium-β-NTA-complex. In addition, higher pH leads to higher concentration of the deprotonated β-NTA^−^ anion (right side of Eq. 1) resulting in more β-NTA^−^ anions available for Fe^3+^ and Eu^3+^ coordination.


1


Therefore, the HEE with a pH of 6.6 favors the quantitative analysis of Eu in the presence of Fe^3+^ ions in comparison with the commercial enhancer (DELFIA^®^), which contains free acetic acid and has a pH of 2.5.

A similar reduction of Eu fluorescence intensity with both enhancer solutions was observed in the presence of Ca^2+^ (data not shown). This is important for the investigation of biological samples, which contain iron, calcium, and other metal ions.

To show that the fluorescence measured actually reflects the Eu-doped nanoparticles (Eu-VSOP) and not free Eu^3+^ ions and to prove that Eu is intercalated into the nanoparticle core, a sample of Eu-VSOP was diluted and filtrated (10 kD filter). The Eu fluorescence of the filtrate was less than 0.1% of the fluorescent signal obtained for the Eu-VSOP solution without filtration (data not shown). This experiment confirms that the fluorescent signal measured for Eu-VSOP-7 originates form the complex formed with β-NTA and TOPO.

Longitudinal comparison of the fluorescence stability developed by both enhancer solutions with Eu-VSOP-7 shows that the HEE develops a longer-lived signal intensity than the one measured with the DELFIA^®^ enhancer, whose signal rapidly decreases (Fig. 4). We speculate that the low pH and the free acetic acid of the DELFIA^®^ enhancer lead to a further release of Eu^3+^ and Fe^3+^ from the nanoparticle core, which might increase the complexation of free iron by the enhancer and decreased Eu fluorescent signal due to saturation of the enhancer.

To meet this concern, the use of HEE for analytical detection of Eu-IONP in solution should be standardized for the sample of interest with a constant time, not shorter than 60 min, between addition of the enhancer and assessment of the Eu fluorescent signal. In addition, data should be normalized to account for variations in the performance of photometers.

### Fluorescence detection of Eu-VSOP in cells and tissue

In this study, we used phagocytic RAW 264.7 macrophages labeled with IONP with the highest content of europium (Eu-VSOP-7) to compare the performance of both enhancer solutions when applied to cells and tissue sections. The RAW 264.7 cells efficiently take up both plain and Europium-doped VSOP.

The effect on PDT suggests that Eu-VSOP-3 and VSOP have slightly better biocompatibility than Eu-VSOP-7 (Additional file 2: Figure S3) in vitro. For future applications, we prefer using Eu-VSOP-4, which has better magnetic characteristics for more sensitive MRI detection as well as better cell biocompatibility than Eu-VSOP-7 (Table 2).

Fluorescence detection of Eu-VSOP was successful in cells and a higher signal stability was achieved using the customized HEE in comparison with the DELFIA^®^ enhancer (Fig. 5). An additional advantage of the customized enhancer is the higher stability of the europium fluorescent signal 2 weeks after storage of cell slides at − 20° (not shown).

In spleen tissue sections, fluorescence detection of Eu-VSOP was achieved for both paraffin-embedded (Fig. 6) and cryosectioned tissue (not shown). The fluorescent signal of Eu-VSOP-7 in spleen sections was robust and proved IONP accumulation in reticuloendothelial structures of splenic red pulp, as seen by conventional Prussian blue staining (Fig. 6a, c).

This result proves the concept for histological detection of Eu-VSOP using fluorescence microscopy. Future studies could include immunofluorescence staining for intracellular and extracellular markers and use genetically modified animals with fluorescent reporters to investigate the cellular and molecular interactions with Eu-VSOP in more detail. Our previous studies using standard VSOP as a tool to investigate neuroinflammation showed evidence of both intra- and extracellular localization of NP in inflamed tissue, although this was difficult to show unambiguously with the limitations of conventional Prussian blue iron staining. With fluorescence detection of Eu-VSOP, confocal microscopy with 3D reconstruction could be used to unambiguously confirm the intracellular or extracellular localization of the particles in both normal and abnormal tissue structures.

In conclusion, we developed stable VSOP doped with europium (Eu-VSOP) and a customized histo-Europium-enhancer (HEE) solution, which, when used in conjunction with Eu-VSOP, produces fluorescent signals that can be detected by fluorescence microscopy in both cells and tissue sections. Furthermore, fluorescence detection of Eu-VSOP provides a stable optical tool to investigate cellular and molecular interactions of Eu-VSOP.

## Additional files

**Additional file 1: Figure S1.** Schematic illustration of the micellar structure formed by free Eu^3+^ and HEE. The europium ion is coordinated by three β-NTA molecules via the two oxygen atoms (red) of the two carbonyl groups and by two TOPO molecules (green) via the oxygen atoms. The complex is surrounded by Triton X-100 forming the micelle.

**Additional file 2: Figure S3.** Effect of nanoparticle uptake on RAW 264.7 macrophages—population doubling time (PDT). Overall, after 4 h incubation (B), macrophages showed a higher uptake of VSOP, followed by Eu-VSOP-3 and Eu-VSOP-7; however, after 24 h incubation (D), average uptake was similar for all NP and ~ tenfold higher. The PDT of labeled and non-labeled cells were compared using two-way ANOVA (n = 3). The PDT of macrophages incubated with NP for 4 h was only significantly (P < 0.001) increased when incubated with Eu-VSOP-7 in comparison to Eu-VSOP-3 and non-labeled cells. However, after 24 h NP incubation, cells labeled with all particles tested—VSOP (P < 0.01), Eu-VSOP-3 (P < 0.001), and Eu-VSOP-7 (P < 0.001)—showed slightly increased PDT in comparison with non-labeled cells. All PDT of labeled NP gradually approached those of non-labeled cells after 6 days when almost all average NP uptake (Fe/cell) was diluted by cell division.

**Additional file 3: Table S1.** Hydrodynamic nanoparticle size measured with dynamic light scattering. Mean hydrodynamic size of VSOP doped with Europium (9.9 to 12.0 nm) is similar to that of nondoped VSOP (10.8 ± 2.8 nm), and there is a narrow distribution of mean diameters (PdI), confirming homogeneity of the synthesized nanoparticles.

**Additional file 4: Figure S2.** Improved buffer capacity of HEE. Fluorescence signal of Eu^3+^ remains stable with HEE solution in acidic conditions. Several Eu^3+^ solutions with c(Eu) = 1000 nmol/L and different HCL in the range from 0.012 to 0.9 mol/L were prepared. 100 μL of these solutions were mixed with 900 μL of the enhancer solution for fluorescence detection. The Eu fluorescence intensity of a solution without HCL was set to 100%. The high buffer capacity of the HEE leads to a stable fluorescence signal with up to 0.5 mol/L of HCL.
