# Supplementary figures and images for: Synthesis of europium-doped VSOP, customized enhancer solution and improved microscopy fluorescence methodology for unambiguous histological detection
Source: J Nanobiotechnology. 2017 Oct 10;15:71. doi: 10.1186/s12951-017-0301-6 (PMC5634840; doi:10.1186/s12951-017-0301-6)

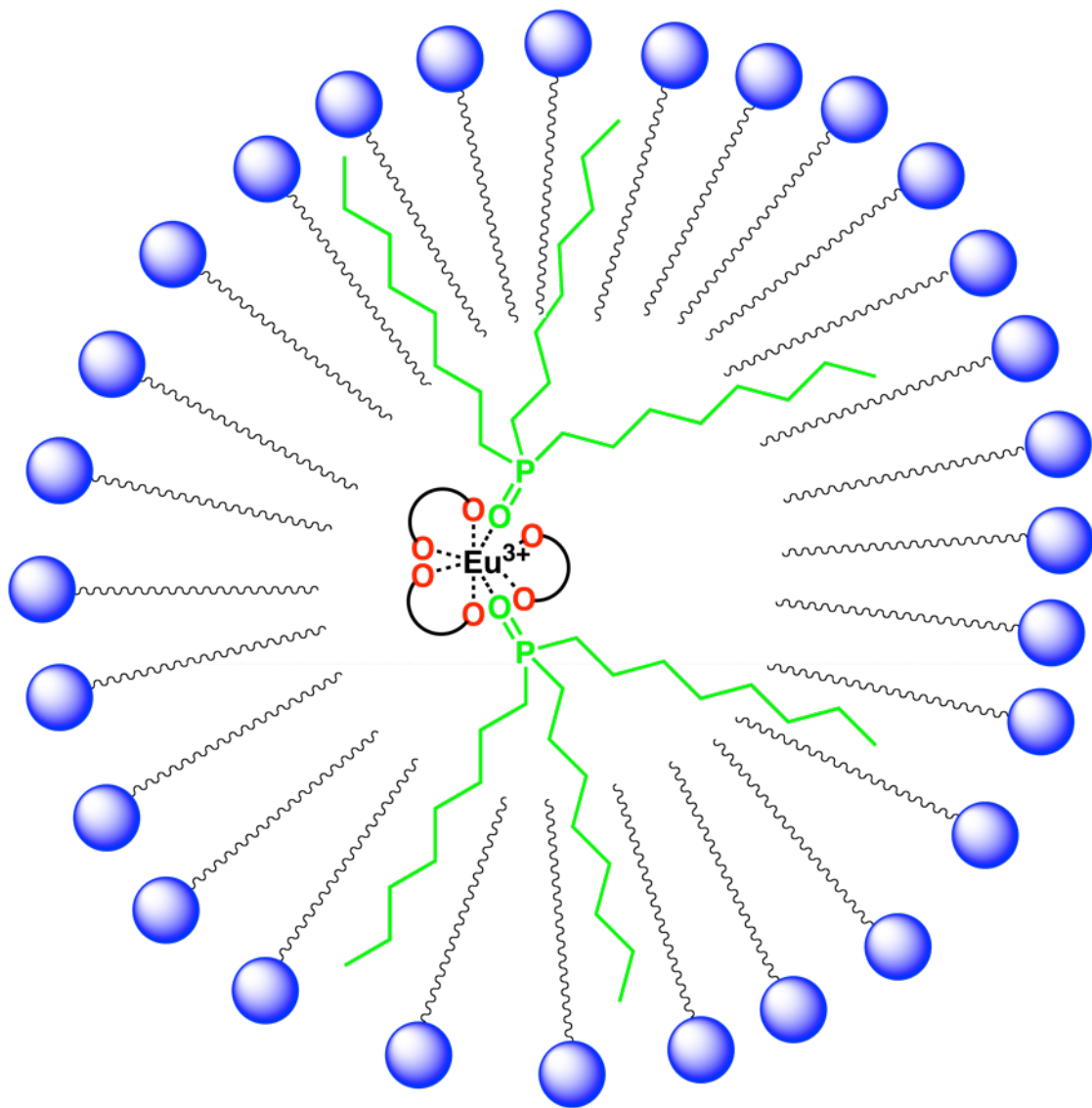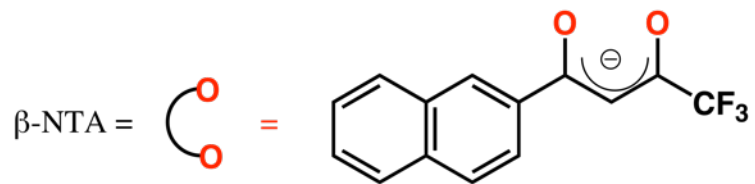

Supplement: Supplementary file 1 — Additional file 1: Figure S1. Schematic illustration of the micellar structure formed by free Eu3+ and HEE. The europium ion is coordinated by three β-NTA molecules via the two oxygen atoms (red) of the two carbonyl groups and by two TOPO molecules (green) via the oxygen atoms. The complex is surrounded by Triton X-100 forming the micelle. [file 12951_2017_301_MOESM1_ESM.pdf]

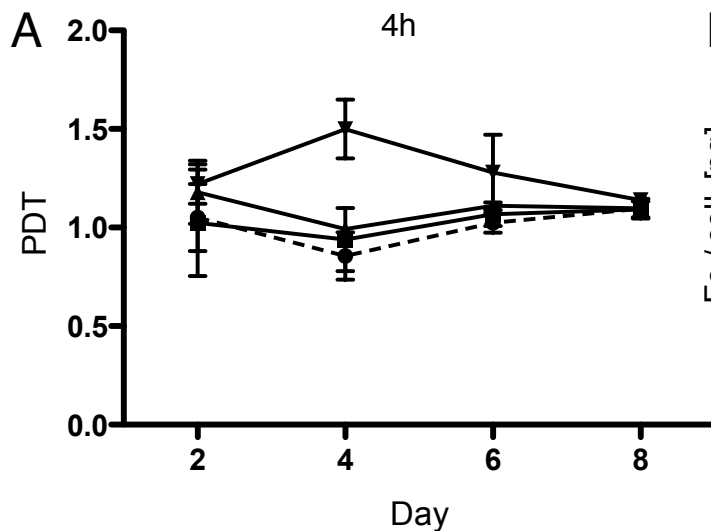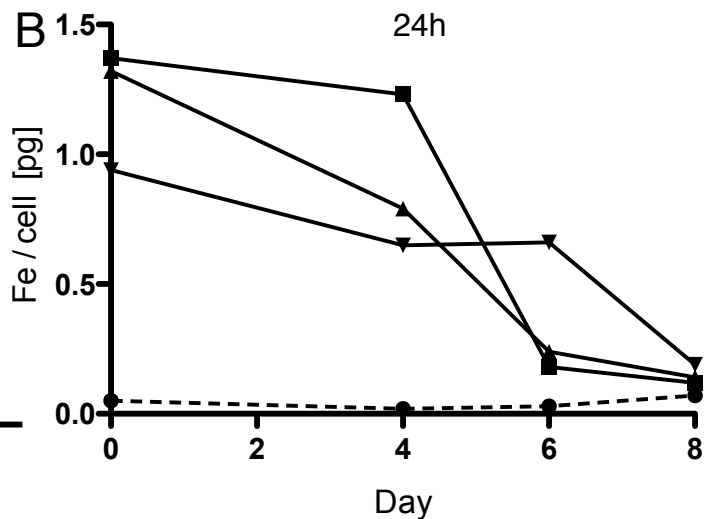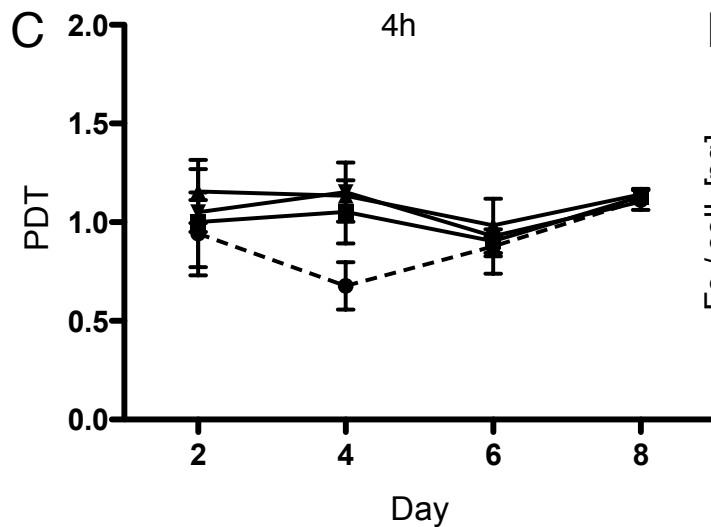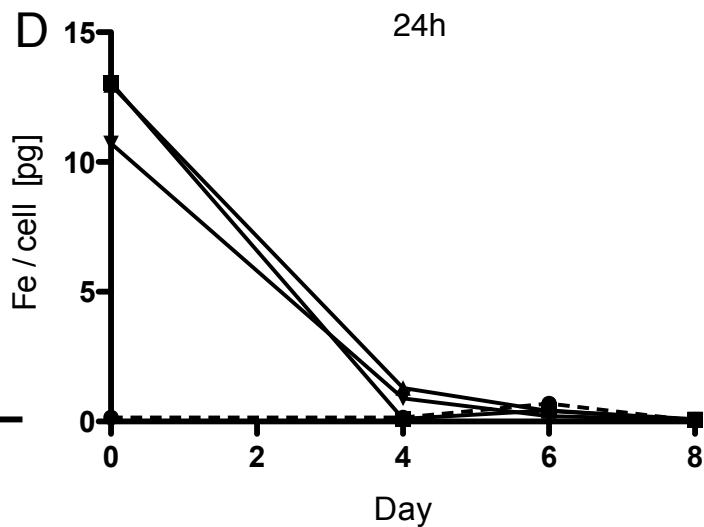

●- - Non labeled      ■ VSOP      ▲ Eu-VSOP-3      ▼ Eu-VSOP-7

Supplement: Supplementary file 2 — Additional file 2: Figure S3. Effect of nanoparticle uptake on RAW 264.7 macrophages—population doubling time (PDT). Overall, after 4 h incubation (B), macrophages showed a higher uptake of VSOP, followed by Eu-VSOP-3 and Eu-VSOP-7; however, after 24 h incubation (D), average uptake was similar for all NP and ~ tenfold higher. The PDT of labeled and non-labeled cells were compared using two-way ANOVA (n = 3). The PDT of macrophages incubated with NP for 4 h was only significantly (P < 0.001) increased when incubated with Eu-VSOP-7 in comparison to Eu-VSOP-3 and non-labeled cells. However, after 24 h NP incubation, cells labeled with all particles tested—VSOP (P < 0.01), Eu-VSOP-3 (P < 0.001), and Eu-VSOP-7 (P < 0.001)—showed slightly increased PDT in comparison with non-labeled cells. All PDT of labeled NP gradually approached those of non-labeled cells after 6 days when almost all average NP uptake (Fe/cell) was diluted by cell division. [file 12951_2017_301_MOESM2_ESM.pdf]

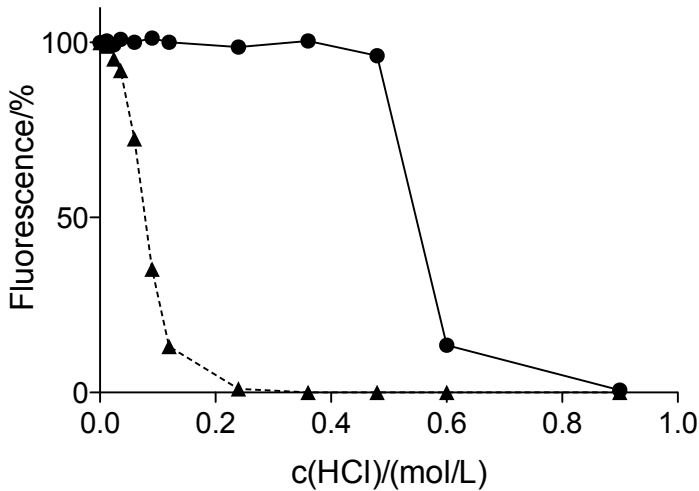

—●— HEE

--▲-- DELFIA® Enhancer

Supplement: Supplementary file 4 — Additional file 4: Figure S2. Improved buffer capacity of HEE. Fluorescence signal of Eu3+ remains stable with HEE solution in acidic conditions. Several Eu3+ solutions with c(Eu) = 1000 nmol/L and different HCL in the range from 0.012 to 0.9 mol/L were prepared. 100 μL of these solutions were mixed with 900 μL of the enhancer solution for fluorescence detection. The Eu fluorescence intensity of a solution without HCL was set to 100%. The high buffer capacity of the HEE leads to a stable fluorescence signal with up to 0.5 mol/L of HCL. [file 12951_2017_301_MOESM4_ESM.pdf]
