# Supplementary material for: Synthesis of europium-doped VSOP, customized enhancer solution and improved microscopy fluorescence methodology for unambiguous histological detection
Source: J Nanobiotechnology. 2017 Oct 10;15:71. doi: 10.1186/s12951-017-0301-6 (PMC5634840; doi:10.1186/s12951-017-0301-6)
Supplement: Supplementary file 3 — Additional file 3: Table S1. Hydrodynamic nanoparticle size measured with dynamic light scattering. Mean hydrodynamic size of VSOP doped with Europium (9.9 to 12.0 nm) is similar to that of nondoped VSOP (10.8 ± 2.8 nm), and there is a narrow distribution of mean diameters (PdI), confirming homogeneity of the synthesized nanoparticles. [file 12951_2017_301_MOESM3_ESM.docx]

Table S1 Hydrodynamic nanoparticle size measured with dynamic light scattering. Mean hydrodynamic size of VSOP doped with Europium (9.9 to 12.0 nm) is similar to that of nondoped VSOP (10.8±2.8 nm), and there is a narrow distribution of mean diameters (PdI), confirming homogeneity of the synthesized nanoparticles.

*Calculated from particle size distribution (hydrodynamic diameter by number).

| VSOP | Z- Average | | Mean diameter/nm* | Particle size distribution  (Hydrodynamic diameter/nm) | PdI |
| --- | --- | --- | --- | --- | --- |
| VSOP | | 15.37 | 10.8±2.8 |  | 0.085 |
| Eu-VSOP-1 | | 14.08 | 10.2±2.5 |  | 0.101 |
| Eu-VSOP-2 | | 15.42 | 10.1±2.7 |  | 0.107 |
| Eu-VSOP-3 | | 16.26 | 9.9±2.8 |  | 0.102 |
| Eu-VSOP-4 | | 14.88 | 10.5±2.8 |  | 0.064 |
| Eu-VSOP-5 | | 16.32 | 11.0±2.9 |  | 0.102 |
| Eu-VSOP-6 | | 19.5 | 12.0±3.4 |  | 0.129 |
| Eu-VSOP-7 | | 17.52 | 11.6±3.1 |  | 0.106 |
